# Prevalence and factors associated with metabolic syndrome in first hospitalization for major depression disorder patients

**DOI:** 10.1038/s41598-023-42720-y

**Published:** 2023-09-19

**Authors:** Zhongyu Tang, Yanping Zhen, Lin Zhang, Xuebing Liu, Jun Ma

**Affiliations:** 1 Department of Psychiatry, Wuhan Mental Health Center, No. 89, Gongnongbing Road, Wuhan, Hubei Province China; 2 Wuhan Hospital for Psychotherapy, Wuhan, China; 3 Department of Psychiatry, The Second Affiliated Hospital of Hubei University of Science and Technology, Xianning, China

**Keywords:** Epidemiology, Depression

## Abstract

Major depressive disorder (MDD) is a common and socially burdensome psychiatric disorder with a causal and complex relationship with metabolic syndrome (MetS), which is often co-morbid. However, the prevalence and risk factors for MetS in patients with MDD are inconclusive. The purpose of this study is to investigate the prevalence and factors influencing MetS in first hospitalization MDD patients. A total of 981 MDD patients were included. Sociodemographic and general clinical data were collected from the patients, while metabolism-related parameters were also measured, and psychological symptoms were assessed. Our study found that the prevalence of MetS in the study population was 9.68%. MDD patients with MetS had higher levels of metabolism-related parameters and more severe psychological symptoms. We identified risk factors for MetS and its severity separately: age of onset of MDD, more severe depressive symptoms, and higher thyroid stimulating hormone (TSH) levels were risk factors for the development of MetS, whereas higher TSH levels were risk factors for the severity of MetS. Our results suggest that MetS is not highly prevalent in MDD patients, but certain risk factors may increase its likelihood and severity, and that these findings could be beneficial for clinical intervention and care of MetS.

## Introduction

Major Depressive Disorder (MDD) is one of the most debilitating chronic mental illnesses^1^, with a 12-month prevalence of about 6% in the population^2^. The life expectancy of people diagnosed with depression is estimated to be shortened by 10–14 years^3^. However, the cause of MDD remains largely unknown, and relapse rates for the disorder remain high^4^. There are indications of a complex relationship between MDD and metabolic syndrome (MetS) that is mutually reinforcing and mutually causal. On the one hand, MDD alters the patient's gut microbiota through the regulatory mechanism of the microbiota-gut-brain axis, which ultimately leads to the development of metabolic disorders such as obesity^5,6^. On the other hand, MetS was found to share genetics and possible risk gene pathways with MDD^7,8^. This implies that MetS may be the native disease of MDD. Clinical studies have also confirmed an increased prevalence of MetS and an increased risk of elevated levels of some metabolic parameters in patients with MDD^9,10^, while the prevalence of depressive disorders in patients with metabolic disorders is equally surprising^11,12^.

The MetS is characterized by the presence of central obesity, hypertension, low levels of high-density lipoprotein cholesterol (HDL-C), elevated triglyceride (TG) levels, and hyperglycemia^13,14^. It is a well-established collection of risk factors for cardiovascular disease (CVD)^15,16^. Consequently, the identification and assessment of MetS risk factors in patients diagnosed with MDD, a population at high risk for MetS, is crucial in clinical practice. In fact, in-depth research on MetS-related studies in MDD populations has been one of the major areas of interest for researchers and psychiatrists. In addition to reporting that MDD and MetS or its components share a common genetic pathway^7,8,17^, researchers have identified important implications of MetS or its components for the MDD population. For example, fasting blood glucose (FBG) levels and HDL-C levels are potential markers used to predict suicide in young MDD patients^18^, low plasma total cholesterol (TC) levels are associated with recent suicide attempts in MDD patients^19^, and TC levels play an important role in the pathophysiology of MDD^20^. All of this informs us of the need for further clarification and understanding of the prevalence and correlates of MetS in the confirmed MDD population.

Although the prevalence of MetS in populations with MDD is currently being addressed by researchers who have reported a high degree of heterogeneity ranging from 20.2 to 45.2%^21–26^, none of these studies have concentrated on the severity of MetS. The objective of this study was to investigate the prevalence of MetS and its associated factors in a larger sample of first hospitalized MDD patients in China. Specifically, we aimed to examine the factors related to the severity of MetS.

## Results

### The differences between clinical subgroups with and without MetS

Out of all the MDD patients included in the study, 94 met the diagnostic criteria for MetS, representing 9.68% (95/981) of the total. There were significant differences in demographic and general clinical data between the subgroups with and without MetS. Specifically, as illustrated in Table 1, the MetS subgroup exhibited higher values in various indicators, including age, onset age, percentage of unmarried individuals, prevalence of suicidal behavior, MetS scores, scores on the four scales (PSS, HAMD, HAMA, and CGI-SI), and TSH, TC, and LDL-C levels, compared to the non-MetS subgroup.

**Table 1:** The demographic and general clinical data in different clinical subgroups.

Index	Total patients (n = 981)	MetS (n = 95)	Non-MetS (n = 886)	$t{/\chi }^{2}$	*p –* value
Age—years	35.62 ± 12.44	44.84 ± 11.25	34.64 ± 12.17	− 8.29	< .001*
Onset age—years	34.09 ± 12.36	43.63 ± 11.07	33.08 ± 12.06	− 8.71	< .001*
Course of disease—months	10.83 ± 4.41	10.36 ± 5.24	10.88 ± 4.32	0.92	0.360
Gender				1.26	0.261
Male	333, 33.94%	27, 28.72%	306, 34.50%		
Female	648, 66.06%	67, 71.28%	581, 66.18%		
Marital status—(n, %)				27.50	< .001*
Unmarried	307, 31.29%	7, 7.44%	300, 33.82%		
Married	674,68.71%	87, 92.55%	587, 73.52%		
Treatment history				2.49	0.115
Yes	636, 64.83%	54, 57.45%	582, 65.61%		
No	345, 35.17%	40, 42.55%	305, 34.39%		
Suicidal behavior				70.16	< .001*
Yes	132, 13.46%	39, 41.49%	93, 10.48%		
No	849, 86.54%	55, 58.51%	794, 89.52%		
Educational background				3.10	0.078
High school and below	683, 69.62%	77, 65.95%	606, 68.32%		
Bachelor and above	298, 30.38%	17, 34.05%	218, 31.68%		
PSS	8.67 ± 4.39	12.86 ± 7.12	8.23 ± 3.73	− 6.22	< .001*
HAMD	29.43 ± 2.97	31.57 ± 3.53	29.21 ± 2.81	− 6.29	< .001*
HAMA	20.28 ± 3.49	22.84 ± 4.15	20 ± 3.3	− 6.42	< .001*
CGI-SI	5.83 ± 0.71	6.17 ± 0.81	5.79 ± 0.69	− 4.36	< .001*
TSH—uIU/mL	3.98 ± 2.47	7.1 ± 3.72	3.65 ± 2.04	− 8.84	< .001*
FT_3_—pmol/L	4.9 ± 0.69	4.83 ± 0.63	4.91 ± 0.7	1.07	0.285
FT_4_—pmol/L	16.78 ± 3.04	16.56 ± 2.95	16.8 ± 3.05	0.75	0.249
MetS scores	0.00 ± 0.35	0.53 ± 0.3	-0.06 ± 0.31	− 17.53	< .001*
MetS components					
WC—cm	79.98 ± 8.4	84.13 ± 8.47	79.55 ± 8.28	− 5.09	< .001*
FBG—mmol/L	5.26 ± 0.63	5.84 ± 0.89	5.2 ± 0.56	− 6.83	< .001*
TG—mmol/L	2.11 ± 1	2.68 ± 1.08	2.05 ± 0.97	− 5.91	< .001*
HDL-C—mmol/L	1.32 ± 0.23	1.21 ± 0.24	1.33 ± 0.22	5.00	< .001*
SBP—mmHg	116.39 ± 11.15	132.22 ± 9.74	114.71 ± 9.9	− 16.33	< .001*
DBP—mmHg	74.62 ± 6.83	84.44 ± 8.57	73.58 ± 5.71	− 12.00	< .001*
TC—mmol/L	4.79 ± 0.92	5.25 ± 0.97	4.74 ± 0.9	− 5.17	< .001*
LDL-C—mmol/L	2.67 ± 0.74	2.84 ± 0.8	2.65 ± 0.73	− 2.43	0.015*
BMI—kg/m^2^	24.18 ± 1.76	24.36 ± 1.8	24.16 ± 1.76	− 1.03	0.302

PSS: Positive symptom subscale; HAMD: Hamilton Depression Scale score; HAMA: Hamilton Anxiety Scale Score; CGI-SI: Clinical Global Impression Scale—Severity of Illness; TSH: Thyroid stimulating hormone; FT_3_: Free triiodothyronine; FT_4_: Free tetraiodothyronine; MetS: Metabolic syndrome; WC: waist circumference; FBG: fasting blood glucose; TG: triglycerides; HDL-C: high density lipoprotein cholesterol; SBP: systolic blood pressure; DBP: diastolic blood pressure; TC: total cholesterol; LDL-C: low density lipoprotein cholesterol; BMI: Body mass index. *p < 0.05.

### Influencing factors of MetS in MDD patients: based on binary logistic model

**Figure 1:**
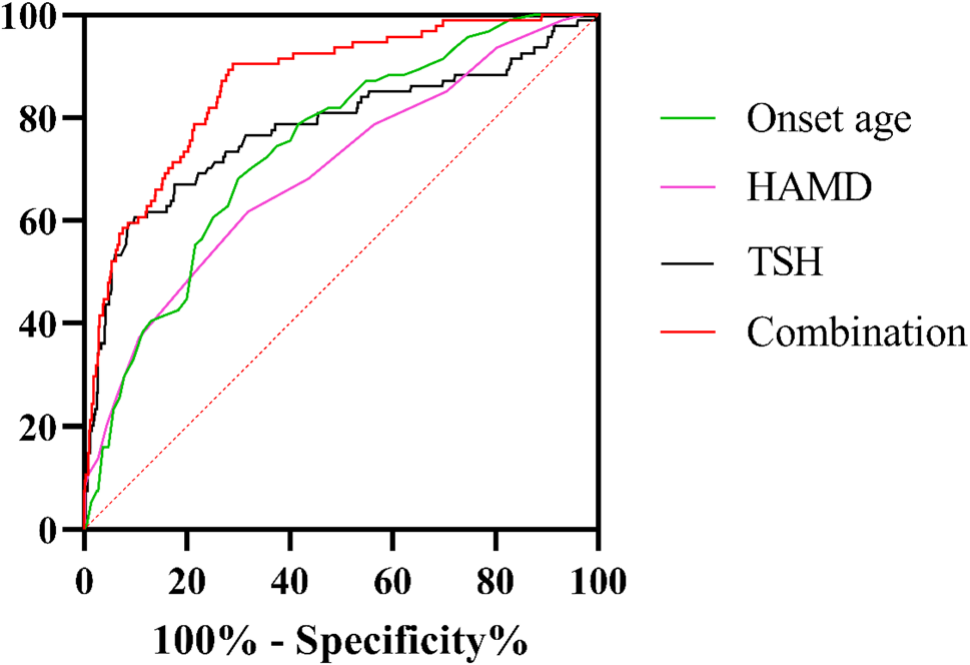
The discriminatory capacity of related factors for distinguishing between patients with and without MetS in MDD patients. The area under the curve of onset age, HAMD score, TSH, and the combination of these three factors were 0.74, 0.69, 0.78, and 0.87, respectively.

Next, we focused on the factors influencing MetS in MDD patients. A binary logistic regression model (Backward: Wald) was constructed with the variables that differed in the univariate analysis as independent variables and MetS as the outcome variable. The results showed that onset age (B = 0.06, *p* < 0.001, OR = 1.06), HAMD scores (B = 0.12, *p* = 0.011, OR = 1.12), and TSH (B = 0.36, *p* < 0.001, OR = 1.44) were risk factors for MetS (Table 2). Moreover, AUCROC revealed the following values for each risk factor: onset age was 0.74, HAMD was 0.69, and TSH was 0.78. To identify MetS from non-MetS, the combination of onset age, HAMD score, and TSH produced a higher AUC value of 0.87 (*p* < 0.001, 95%CI = 0.83–0.90) (Fig. 1).

**Table 2:** Binary logistic regression analyses of determinants of MetS in MDD patients.

	Coefficients	Std. error	Wald	*p*-value	95% CI for EXP (B)		
	B				Exp(B)	Lower	Upper
Constant	-9.72	1.39	48.78				
Onset age—years	0.06	0.01	21.03	< .001*	1.06	1.04	1.09
Marital status (unmarried *vs.* married)	0.83	0.49	2.90	0.089	2.29	0.88	5.93
HAMD	0.12	0.05	6.41	0.011*	1.12	1.03	1.23
TSH—uIU/mL	0.36	0.04	66.95	< .001*	1.44	1.32	1.57
LDL-C—mmol/L	-0.33	0.18	3.35	0.067	0.72	0.51	1.02

HAMD: Hamilton Depression Scale score; TSH: Thyroid stimulating hormone; LDL-C: low density lipoprotein cholesterol. *p < 0.05.

### Influencing factors of MetS scores in MDD patients: a multiple linear regression model

Finally, we construct a multiple linear regression model (Input) with MetS as the outcome variable and the relevant factors affecting MetS in the previous step as the dependent variables. The results showed that TSH (B = 0.03, t = 3.23, *p* = 0 0.002, 95%CI 0.01–0.02) was risk factors for higher MetS scores (Table 3).

**Table 3:** Correlates affecting MetS scores in MDD patients: a multiple linear regression model.

	Coefficients	Std. error	*t*	*p-*value	95% CI	
	B				Lower	Upper
Constant	0.21	0.29	0.73	0.470	− 0.36	0.78
Onset age	0.00	0.00	− 0.34	0.735	− 0.01	0.00
HAMD	0.01	0.01	0.58	0.563	− 0.01	0.02
TSH	0.03	0.01	3.23	0.002*	0.01	0.05

HAMD: Hamilton Depression Scale score; TSH: Thyroid stimulating hormone. *p < 0.05.

## Discussion

To the best of our knowledge, this is the only study that reports factors related to the severity of MetS in MDD patients in Chian. The main findings of our study are as follows: 1. The prevalence of MetS in the included group was 9.68%. 2. Compared to the non-MetS subgroup, the MetS subgroup had not only higher levels of a wide range of metabolic parameters, but also more severe psychopathological and psychological symptoms, such as scores on the four scales of PSS, HAMD, HAMA, and CGI-SI, and higher age and onset age of the patients. 3. Onset age, HAMD scores, and TSH levels were risk factors for the diagnosis of MetS. 4. The levels of TSH was risk factor for higher MetS scores.

Several factors contribute to the substantial heterogeneity in the prevalence of MetS among individuals diagnosed with MDD, including variations in ethnicity^27^, geography^10^, and distinct diagnostic criteria for MetS^13,28^. In our study, we reported a MetS prevalence of 9.68% among MDD patients, which is significantly lower than the figures observed in similar research. Focusing solely on the East Asian population, a small sample from Taiwan, China, showed a 34.3% prevalence of MetS^21^, while a large sample from Japan indicated a 14.0% prevalence^29^. Although the MetS prevalence reported in these two studies differs greatly, both percentages were notably higher than those documented in our research. Upon comparison, we discovered that both studies used the same diagnostic criteria for MetS, but their criteria were more stringent than ours, which may account for the marked difference in reported prevalence. In contrast, a recently published national epidemiological survey from China reported a standardized prevalence of MetS of 31.1% among the general adult population in China^30^. This prevalence is roughly comparable to that reported in Taiwan, China, which has the highest prevalence of MetS in MDD patients^21^. In conclusion, we determined that the prevalence of MetS was relatively low among first-time hospitalized MDD patients in China.

MDD is often regarded as an independent risk factor for MetS^31^, primarily due to the shared genetic risk pathway between the two conditions^7^. However, does this imply that increased severity of depressive symptoms correlates to a higher risk of MetS diagnosis? Several studies have provided affirmative answers to this question^22,32,33^, consistent with our findings. Another study reported a positive correlation in females, but an inverse relationship in males^34^. Further research indicated that the severity of depressive symptoms is associated with specific components of MetS, such as elevated blood glucose levels, higher TG levels, lower HDL-C levels, and increased WC^35,36^. Generally, both MDD and depressive symptom severity are important independent risk factors for MetS comorbidity in MDD patients. Higher TSH levels have previously been identified as significant risk factors for MetS in non-MDD populations^37,38^, and our study expands the range of populations for which these findings apply. Additionally, we report that increased onset age of MDD is the third risk factor for MetS diagnosis in MDD patients. While we have not found similar reports to date, age is a crucial contributor to MetS diagnosis in the general population^30^, and MDD itself is a risk factor for MetS^31^. Consequently, we consider the age of MDD onset as a specific result of the dual influence of age and MDD on MetS. In further ROC analysis, we determined that the triad of onset age of MDD, HAMD scores and TSH levels had good combination diagnostic capability for MetS.

Finally, we report higher TSH levels as a factor influencing the severity of MetS. Up to now, there are relatively few studies on factors related to the severity of MetS in the MDD population. The limited number of studies have reported a large heterogeneity of study objectives or outcomes. For example, one study prospective study reported a significant prospective association between initial depressive symptoms and subsequent MetS scores among clergy^39^. Another study found that in the African American female population, higher depressive symptom scores were associated with higher MetS severity in women^40^. Unfortunately, in the present study our findings differ from the two aforementioned studies in that we report no significant effect of the severity of depressive symptoms on the severity of MetS. Whether the reason for this is due to ethnicity, geography or sampling error needs to be further investigated and verified in the future. As mentioned earlier, TSH is an important risk factor for MetS even in the MDD population. Studies have shown that the development of MetS^41,42^, and related parameters of MetS, such as weight gain^43^, are positively correlated with TSH levels. It has also been reported that the prevalence of MetS, abdominal obesity and hypertriglyceridemia is higher in subjects with clinical hypothyroidism^44^. Although none of these studies are direct evidence that higher TSH levels are a risk factor for MetS severity, they are certainly the strongest supporting evidence.

The present study also has several limitations. First, as a cross-sectional study, our results cannot clarify the causal relationship between MetS, its severity and influencing factors. This requires further prospective studies. Second, our sample consisted of patients in the acute phase requiring hospitalization, so our findings may not be generalizable to MDD patients in the symptom-stable phase. Third, our sample included both drug-naive samples and samples with a history of outpatient treatment, which increased the confounding factors of this study, and we will report separately on the effect of antidepressant exposure on the results. Fourth, due to the relatively small number of MetS subgroup cases we actually obtained, this may lead to limitations in the further generalization of the results obtained from our regression analysis, larger sample sizes may be beneficial in addressing this issue. In the future, we will try to properly control for the above shortcomings.

In conclusion, the prevalence of MetS in MDD patients is relatively low. Risk factors for MetS include early onset of MDD, more severe depressive symptoms, and elevated levels of TSH. Moreover, higher TSH levels also contribute to the severity of MetS. Identification of these risk factors has proven valuable for psychiatrists in the evaluation and detection of MetS and its severity in patients with MDD during their initial hospitalization. This knowledge helps in guiding interventions and implementing appropriate care measures.

## Materials and methods

### Subjects

A total of 1012 patients with MDD who were first hospitalized at Wuhan Mental Health Center from July 2017 to August 2022 were included, of which 3 were pregnant or gestational, 7 had a history of substance abuse, 3 co-morbid personality disorders, 18 co-morbid diabetes mellitus, and finally 981 were included in the statistical analysis.

Patients were eligible to meet the following inclusion criteria:

Meet the diagnostic criteria for MDD in the 10th revision of the International Classification of Diseases (ICD-10).
There was no history of hospitalization before the inpatient interview that day.
Aged 18–60 years old, Chinese Han nationality.
4.Their 17-item Hamilton Depression Scale (HAMD-17) total score needed to be ≥ 24.

Patients who meet one condition will be excluded:

1. Breast-feeding patients are pregnant women.
2. They have a history of material dependence.
3. Patients with serious physical diseases or personality disorders.
4. Patients with a clear history of diabetes mellitus in the past.
5. Patients who are unable to cooperate with psychological symptom evaluation for any reason.

The study was reviewed and approved by the Ethics Committee of Wuhan Mental Health Center, and all participants signed a written informed consent form. All participants have written informed consent signed by the patient himself or his family. Patients have the right to withdraw this study at any time. All methods were performed in accordance with the relevant guidelines and regulations.

### Research design

This study was designed as a cross-sectional study. For the included MDD patients with initial hospitalization, we first calculated their prevalence of MetS, compared the differences in demographics and general clinical treatment between the two clinical subgroups with and without MetS, analyzed the factors associated with MetS, and the factors associated with MetS scores.

For MDD patients who met the inclusion criteria, we completed the collection of general clinical data on the day of the patient's visit, including age, gender, age of onset, course of disease, marital status, whether accompanied by suicidal behavior, and whether there is a history of outpatient treatment. At the same time, the patient's venous blood was collected to detect the patient's blood lipid profile (specifically: total cholesterol, TC; triglycerides, TG; low density lipoprotein cholesterol, LDL-C; high density lipoprotein cholesterol, HDL-C) level, fasting blood glucose (FBG) level, body mass index (BMI), blood pressure level (specifically: systolic blood pressure, SBP; diastolic blood pressure, DBP), and thyroid function (specifically: thyroid stimulating hormone, TSH; free triiodothyronine, FT_3_: Free triiodothyronine; FT_4_: free tetraiodothyronine) level. The severity of depressive symptoms was assessed using the Hamilton Depression Scale (HAMD-17), the severity of anxiety symptoms was assessed using the Hamilton Anxiety Scale (HAMA-14), the severity of psychotic symptoms was assessed using positive symptom subscale (PSS, a subscale of the Positive and Negative Symptom Scale, containing 7 items, items P1-P7, respectively), and the severity of pre-treatment illness was assessed using the Clinical Global Impression Scale (CGI).

Diagnostic criteria of metabolic syndrome: the diagnostic criteria for metabolic syndrome in China require that at least three of the following five indicators be met^45^: 1. abdominal obesity: waist circumference (WC) ≥ 90 cm in men and ≥ 85 cm in women. 2. hyperglycemia: FBG ≥ 6.1 mmol/L and/or those who have been diagnosed and treated for diabetes mellitus. 3. hypertension: SBP ≥ 130/85 mmHg or DBP ≥ 85 mmHg or confirmed and treated hypertension. 4. TG ≥ 1.70 mmol/L. 5. HDL-C < 1.04 mmol/L.

Scoring rules for MetS: based on previous studies, we have scored the severity of the MetS in including patients of the MetS^46,47^. According to the scoring rules, we first calculated the reciprocal of HDL-C and the mean arterial pressure (MAP) using the formula MAP = $1/3\times SBP+2/3\times DBP$. Following this, we normalized the five MetS parameters: waist circumference (WC), triglycerides (TG), the reciprocal of HDL-C, fasting blood glucose (FBG), and MAP. Next, we performed a principal component analysis with varimax rotation on the five normalized components to derive principal components (PCs) with an eigenvalue of 1.0 or higher, which accounted for a substantial portion of the observed variation. In this study, PC1 and PC2 explained 25.23% and 20.85% of the variance, respectively [loadings PC1 (PC2): WC 0.26 (− 0.63), TG 0.28 (0.50), HDL-C 0.17 (0.61), MAP 0.73 (0.04), and FBG 0.75 (− 0.15)]. Finally, the weighted PC scores were determined by the relative weights of PC1 and PC2 in the explained variance. To obtain the MetS score, we added up the individual weighted PC scores.

The assessment of the relevant psychological scales was done by 2 uniformly trained psychiatrists with the title of attending or higher, belonging to the medical institution of the sample source.

### Data analysis

The categorical variables are stated in terms of counts, while the data acquired for the normally distributed continuous measures are reported in terms of mean and standard deviation. T-tests on independent samples were employed to compare continuous variable from various groups. Chi-squared tests was used to compare rates. Then, the variables that differed in the univariate analysis were included in a binary logistic regression model as independent variables, with MetS as the dependent variable, to analyze the factors influencing MetS. The area under the receiver operating characteristics (AUCROC) was used to determine the discriminatory capacity of significant parameters to distinguish between patients with and without MetS. Finally, a multiple linear regression model was constructed with the MetS score as the outcome variable and the factors influencing MetS in binary logistic regression as the independent variables to determine the factors influencing the severity of MetS. All *p* values were 2-tailed, and the significance level was < 0.05. Statistical analyses were performed using SPSS 27 (SPSS, Inc., Chicago, IL).

## Data Availability

The datasets used and/or analyzed during the current study available from the corresponding author on reasonable request.
